# Longitudinal modelling of growth in neonates exposed to antenatal steroids to quantify associations with final height: a cohort study

**DOI:** 10.1136/archdischild-2025-329091

**Published:** 2025-07-22

**Authors:** Neil Richard Lawrence, Krish Panchigar, Simon J Clark, Tim J Cole, Gary S Collins, Jeremy F Dawson, Nils P Krone, Neil Wright

**Affiliations:** 1 Division of Clinical Medicine, The University of Sheffield—Western Bank Campus, Sheffield, UK; 2 Division of Computer Science, The University of Sheffield—Western Bank Campus, Sheffield, UK; 3 Jessop Wing, Neonatal Unit, Sheffield Teaching Hospitals NHS Foundation Trust, Sheffield, UK; 4 Institute of Child Health, University College, London, UK; 5 NDORMS, University of Oxford, Oxford, UK; 6 Institute of Work Psychology, Management School, The University of Sheffield—Western Bank Campus, Sheffield, England, UK; 7 Academic Unit of Child Health, Department of Oncology and Metabolism, University of Sheffield, Sheffield, UK; 8 Paediatric Endocrinology & Diabetes, Sheffield Children’s Hospital, Sheffield, UK

**Keywords:** Growth, Intensive Care Units, Neonatal, Neonatology, Statistics, Infant Development

## Abstract

**Objective:**

To assess the associations of antenatal steroids with child growth.

**Design:**

Longitudinal observational cohort study started in 1994.

**Setting:**

A single tertiary neonatal centre in Sheffield, UK.

**Participants:**

Of 254 individuals recruited, two were excluded, 48 born at term; 202 (57% boys, 87% white ethnicity) modelled had a median of 19 height measurements each (Q1:12 to Q3:21) up to median age 15.8 years (Q1:9.9 to Q3:16.9).

**Interventions:**

Data on administration of antenatal steroids were collected alongside gestational age and parental height.

**Main outcome measures:**

Height was modelled with SuperImposition by Translation and Rotation (SITAR) to extract each person’s peak velocity and age at peak velocity via the SITAR random effects of ‘size’, ‘timing’ and ‘intensity’ and to predict height at 18 years. The association of each random effect and final height with exposure to antenatal steroids was assessed by multiple regression to adjust for covariates.

**Results:**

In girls with covariates available (n=59/87), exposure to antenatal steroids was positively associated with SITAR ‘size’ and ‘intensity’ of growth when adjusted for gestational age, maternal and paternal height, equating to a final height 2.8 cm (95% CI 0.3 to 5.3 cm) greater than for those not exposed to antenatal steroids. In boys (n=66/115), exposure to antenatal steroids had no association with final height.

**Conclusions:**

This observational cohort study showed greater height of girls exposed to antenatal steroids not seen in boys. Analysis of existing long-term follow-up data from neonates is indicated to increase understanding of the associations of neonatal interventions on growth.

**WHAT IS ALREADY KNOWN ON THIS TOPIC**

- Babies born prematurely tend to be shorter as adults, the deficit increasing with greater prematurity.
- Antenatal steroids reduce the risk of early morbidity and mortality, but their associations with later child growth are less clear.

**WHAT THIS STUDY ADDS**

- This longitudinal observational cohort study showed 2.8 cm larger adult height (95% CI 0.3 to 5.3 cm) in girls treated with antenatal steroids in comparison to those not exposed to antenatal steroids, after controlling for gestation and parental height. There was no observed increase in boys.

**HOW THIS STUDY MIGHT AFFECT RESEARCH, PRACTICE OR POLICY**

- Further research is needed to investigate sex differences in childhood growth after exposure to antenatal steroids.

## Introduction

 Preterm birth is the main cause of neonatal mortality and morbidity, with more than one in 10 babies born before 37 weeks gestation.^1^ Earlier gestation is associated with a greater risk of a poor outcome, alongside fetal growth restriction, male sex and multiple pregnancy.^2^ Despite improvements in both obstetric and neonatal care, neonatal disorders remain the largest source of disability-adjusted life years worldwide, surpassing that of any single-organ disease of adulthood, including ischaemic heart disease.^3^

Outcomes for babies born before 35 weeks gestation are improved if glucocorticoids are administered to the mother in the week preceding birth.^4^ Antenatal steroids were found to be effective to reduce rates of respiratory distress syndrome as early as 1972,^5^ yet a decade later physicians using them routinely remained in the minority, before meta-analyses helped establish their use as the gold standard.^6 7^

Short-term benefits of antenatal steroids in prematurity extend beyond the lungs to reduced rates of intraventricular haemorrhage, necrotising enterocolitis and overall mortality. However, there have recently been concerns about increased interventions and long-term adverse neurocognitive outcomes in babies exposed to antenatal steroids born at full term,^8 9^ highlighting the need for ongoing research to optimise the use of antenatal steroids and better inform patients about the risks and benefits. Babies born preterm are shorter than their peers born at term, with increasing deficit at earlier gestations.^10 11^ Previous studies have shown no impact on height of exposure to antenatal steroids,^7 12^ although several studies following preterm babies to adulthood have not adjusted for gestational age^13 14^ or parental height, both of which affect final height.^15^

Significant differences in outcomes between boys and girls have been demonstrated from early in gestation, with boys more likely to be stillborn or born preterm than girls,^16^,^18^ more likely to suffer complications related to oxidative stress and significant lung disease,^19^ and more frequently displaying cardiovascular risk factors at later ages.^1^ However, there is uncertainty about any differences in outcomes related to the administration of antenatal steroids with sex.^20 21^ This study uses growth curve modelling of a historic cohort to assess longitudinal data in neonates and investigate associations of exposure to antenatal steroids with childhood growth trajectories in each sex.

## Methods

This was a single-centre observational cohort study (Sheffield, UK) of individuals recruited between 1994 and 1995 and following them from birth to adult height. The cohort has been used previously to quantify the impact of prematurity on childhood growth.^11^ The infants were born before 37 weeks gestation and admitted to the neonatal unit. Exclusion criteria were living more than 20 miles from the study centre, or suspicion of early severe neurological impairment following cranial ultrasound findings or clinical condition at discharge. Term babies were recruited from the postnatal ward of the same hospital using stratified sampling targeting proportional representation of sex and rates of maternal smoking.^11^

Treatment with antenatal glucocorticoids for expected preterm labour was extracted from clinical records. Maternal and paternal height was measured using a portable stadiometer (Leicester Height Measure) while they were in hospital. Length of participants was quantified with a PedoBaby ruler in the neonatal unit and height by the Leicester Height Measure after 2 years of age. Individuals were measured weekly to 8 weeks, at 8 and 12 months, biannually to age 5 and annually thereafter, until either 18 years or height velocity <1 cm/year, whichever was sooner.

### Statistical methods

#### Height modelling

Individuals with three or more measurements were included in the analysis. Height was modelled on the natural logarithm of exact post conceptual age using the growth curve model SuperImposition by Translation and Rotation (SITAR)^22^ in *R: A Language and environment for statistical computing*.^23^ This is a multilevel model assessing repeated measurements in participants by estimating three participant-specific random effects and a mean natural cubic B-spline curve. The first random effect allows for translation on the Y axis (the ‘size’ of the participants), the second for translation on the X axis (the ‘timing’ of growth) and the third for rotation of each curve (the ‘intensity’ of growth)—see online supplemental appendix 1. Males and females were modelled separately. The optimal spline degrees of freedom for each sex was identified by minimising the Bayesian Information Criterion.

### Outcome metrics

Six outcomes for each participant were extracted from the fitted SITAR models: the random effects of ‘size’, ‘timing’ and ‘intensity’, peak height velocity, age at peak and final height as predicted from the model at 18 years.

### Multivariable modelling

The association of antenatal steroids with growth of participants was assessed controlling for parental height and gestational age at delivery using multiple linear regression. Missing values for covariates were considered missing completely at random with models estimated using a complete case analysis. A p value of 0.05 was used to define statistical significance. The association of antenatal steroids without adjusting for covariates was estimated for comparison.

## Results

### Study participants and measurements

**Figure 1:**
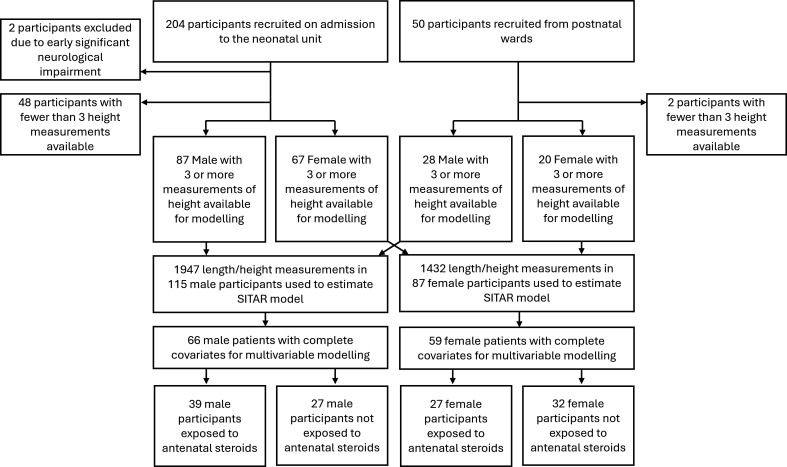
Study recruitment and data availability for modelling. SITAR, SuperImposition by Translation and Rotation.

A total of 254 infants were recruited between 1994 and 1995, 50 of whom were born at term. Two were later excluded due to early significant neurological impairment. There were 202 infants (57% boys, 87% white ethnicity) with three or more height measurements available for modelling (table 1, figure 1). Participants were followed to median age 15.8 years (Q1: 9.9 to Q3: 16.9), 1947 measurements in boys and 1432 in girls (median 19 per participant, Q1: 12 to Q3: 21).

**Table 1:** Demographics of cohort

Sex	Boys		Girls	
Administered antenatal steroids:	Yes	No	Yes	No
n (%) missing	47 (57%)	36 (43%)	32 (46%)	38 (54%)
	n=32		n=17	
Gestational age at delivery (weeks) median (Q1–Q3)	31.1 (29.4–32.9)	35.9 (34.4–40.6)	32.5 (30.0–33.7)	36.3 (34.5–40.3)
Maternal height (cm) median (Q1–Q3) missing	160.0 (155.3–165.5) n=8	160.8 (157.6–166.6) n=7	160.6 (156.8–170.0) n=4	161.6 (158.3–166.5) n=5
Paternal height (cm) median (Q1–Q3) missing	180.0 (170.8–185.0) n=8	175.0 (169.8–182.8) n=9	179.0 (171.0–183.0) n=5	177.5 (173.5–183.5) n=5
Ethnicity: n (%)				
White	42 (90%)	31 (86%)	29 (91%)	36 (95%)
Black	1 (2%)	0 (0%)	1 (3%)	0 (0%)
Asian	2 (4%)	0 (0%)	0 (0%)	1 (3%)
Arab	1 (2%)	2 (6%)	0 (0%)	0 (0%)
Mixed	1 (2%)	3 (8%)	2 (6%)	1 (3%)

Q1, first quartile; Q3, third quartile.

### Missing data and attrition from study

**Figure 2:**
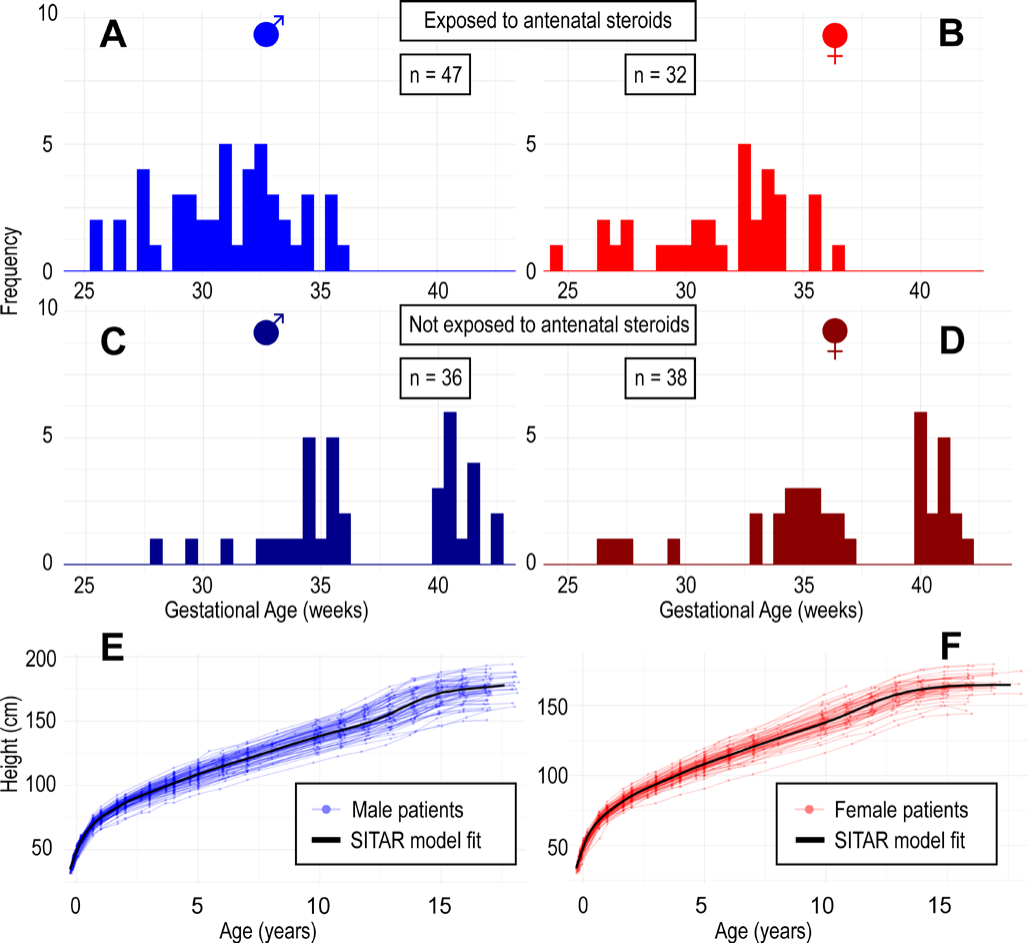
Distribution of gestational age at birth and SITAR height models. (A) Male participants exposed (to antenatal steroids); (B) female participants exposed; (C) male participants not exposed; (D) female participants not exposed; (E) SITAR model fixed effects estimated from all male participants (rendered over raw data); (F) SITAR model fixed effects estimated from all female participants. SITAR, SuperImposition by Translation and Rotation.

The proportion of participants remaining in the study fell over time from 181/202 (90%) at 2 years to 148/202 (73%) at 10 years and 79/202 (39%) at 16 years. Attrition was similar between the sexes and between exposed and unexposed to steroids. Data on gestational age and sex were complete (figure 2), while antenatal steroid status, maternal height and paternal height were missing in 24%, 19% and 20% of patients, respectively (online supplemental appendix 2). Those who received antenatal steroids varied by number of doses administered: 37/66 received one, 17/66 two and 9/66 three or more (3/66 missing).

### SITAR modelling

The optimal model used 12 degrees of freedom for girls (n=87, root mean squared error (RMSE)=1.3 cm), and 15 degrees of freedom for boys (n=115, RMSE=1.5 cm). The mean SITAR growth curves and individual raw curves are presented in figure 2. Summaries of the random effects for participants grouped by steroid exposure status along with peak height velocity, age at peak velocity and final height are given in table 2.

**Table 2:** Unadjusted height metrics derived from SITAR models by exposure to antenatal steroids status

Sex	Boys		Girls	
Exposure to antenatal steroids	No	Yes	No	Yes
Final measured height (cm) mean (SD) number remaining in study	178.2 (7.8) n=9	172.6 (9.8) n=18	162.6 (6.2) n=25	164.6 (8.6) n=17
SITAR† derived growth parameters:	n=85		n=70	
Height at 18 years (cm) mean (95% CI)	180.4 (166.1 to 194.7)	178.5 (161.8 to 195.2)	164 (154.6 to 173.8)	165.4 (153.8 to 177.0)
Peak height velocity (cm/year) mean (95% CI)	9.2 (7.2 to 11.2)	9.2 (7.4 to 11.0)	7.5 (6.1 to 8.9)	8.0 (6.2 to 9.8)
Age at peak height velocity (years) mean (95% CI)	13.0 (10.8 to 15.2)	12.9 (11.1 to 14.7)	11.2 (9.4 to 13.0)	10.8 (8.8 to 12.8)
SITAR patient-level random effects:				
'Size' mean (95% CI)	0.7 (−8.7 to 10.1)	−1.2 (−14.9 to 12.5)	−0.3 (−10.1 to 9.5)	0.9 (−10.7 to 12.5)
'Timing' mean (95% CI)	0.00 (−0.12 to 0.12)	−0.01 (−0.19 to 0.16)	0.00 (−0.15 to 0.15)	0.01 (−0.17 to 0.18)
'Intensity' mean (95% CI)	0.00 (−0.10 to 0.11)	0.00 (−0.13 to 0.13)	−0.02 (−0.11 to 0.08)	0.02 (−0.09 to 0.13)
SITAR model fit statistics:				
Root mean squared error (cm)	1.5		1.3	

* Final height was defined as height when growth velocity <1cm/year or height after 18 years.

† One SITAR model estimated separately in male and female. Parameters were then summarised for participants within each group dependent upon exposure to antenatal steroids.

SITAR, SuperImposition by Translation and Rotation.

### Multivariable modelling

Each modelled outcome metric provided six models for each sex, interpretation concentrating on the regression coefficient for exposure to antenatal steroids. Missing data on covariates allowed the multivariable modelling of 66/115 boys and 59/87 girls.

In boys, steroid exposure was not observed to be related to any outcome (table 3, online supplemental appendix 3). In girls, steroid exposure was statistically significantly associated to predicted height at 18 years, with exposed participants 2.8 cm taller than those unexposed, after controlling for parental height and gestational age. The effect of steroid exposure on age and height velocity of the peak growth spurt were not statistically significant. SITAR size and intensity in girls were significantly related to steroid exposure, the size effect of 2.9 cm closely matching that for final height, while the intensity effect of 3.5% indicated faster growth throughout childhood.

**Table 3:** Adjusted and unadjusted estimates of association of antenatal steroid exposure with SITAR derived height metrics

Dependent variable:	Boys			Girls		
	Independent variable: exposure to antenatal steroids: yes regression estimate (95% CI)		Model R² (adjusted | unadjusted)	Independent variable: exposure to antenatal steroids: yes regression estimate (95% CI)		Model R² (adjusted | unadjusted)
Metrics calculated from modelling:	Adjusted for covariates*	Unadjusted		Adjusted for covariate exposure to antenatal steroids: yes regression estimates*	Unadjusted	
Height at 18 years (cm)	−3.8 (−8.9 to 1.3)	−2.0 (−5.5 to 1.6)	0.36 | 0.01	**2.8 (0.3 to 5.3)**	1.1 (−1.5 to 3.7)	0.56 | 0.01
Peak height velocity (cm/year)	−0.5 (−1.2 to 0.2)	0.0 (−0.5 to 0.4)	0.17 | 0.01	0.4 (−0.2 to 0.9)	**0.5 (0.1 to 0.9)**	0.24 | 0.09
Age at peak height velocity (years)	0.4 (−0.3 to 1.1)	−0.1 (−0.5 to 0.3)	0.07 | 0.01	−0.1 (−0.8 to 0.5)	−0.4 (−0.9 to 0.1)	0.08 | 0.04
SITAR patient-level random effects:						
Size (cm)	−1.0 (−4.8 to 2.7)	−2.0 (−4.7 to 0.7)	0.44 | 0.03	**2.9 (0.4 to 5.4)**	1.2 (−1.4 to 3.8)	0.55 | 0.01
Timing (%)	1.7 (−3.4 to 6.7)	−1.3 (−4.8 to 2.2)	0.38 | 0.01	3.0 (−2.0 to 8.0)	0.9 (−3.0 to 4.9)	0.24 | 0.01
Intensity (%)	−1.8 (−5.5 to 1.9)	−0.7 (−3.3 to 2.0)	0.40 | 0.01	**3.5 (1.0 to 6.0)**	**3.4 (0.9 to 5.8)**	0.55 | 0.10

Statistically significant estimates highlighted in bold. The raw model coefficient for timing and intensity is multiplied by 100 to represent percentage difference between patients exposed and not exposed to steroid. Positive timing represents a later pubertal growth spurt, positive intensity representing a compressed, or faster, pubertal growth spurt.

SITAR, SuperImposition by Translation and Rotation.

Raw data on final measured height in the study were also modelled with covariates (online supplemental appendix 3) and showed a similar mean effect size in height at 18 years and SITAR size, although this was not statistically significant due to the reduced cohort that remained in the study all the way through to a final measurement.

## Discussion

This study used SITAR growth curve modelling^22^ to assess the association between antenatal steroids exposure and the growth trajectory of preterm infants. The findings showed a significantly greater final height in exposed girls after controlling for gestational age and parental height, along with greater SITAR size and faster growth intensity effects through childhood. Boys showed no significant differences.

Several studies have assessed growth of preterm infants to adult height, with strong evidence that premature infants are shorter than term infants at each stage of development,^1^ with variable degrees of ‘catchup growth’.^11 24^ Nonetheless, there is an appreciation that those born at earlier gestations and of extremely low birth weight do not achieve the same final height as their peers born at term.^1^

The short-term benefits of antenatal steroids in babies at the earliest survivable gestations are well recognised, improving mortality and reducing the incidence of respiratory distress syndrome, intraventricular haemorrhage and necrotising enterocolitis.^2 7^ However, there are concerns that antenatal steroid exposure in babies born at term may be detrimental in terms of their requirements for neonatal intensive care and poorer long-term neurocognitive outcomes.^1 8^ While adverse neurocognitive outcomes have been associated with postnatal courses of dexamethasone, lower dose hydrocortisone in preterm infants has been shown not to adversely affect neurodevelopment.^25^ Understanding of the optimum dose, timing and duration of steroids in prematurity continue to evolve.

The greater risk of mortality and complications related to oxidative stress and significant lung disease in boys is highlighted within UK clinical guidelines as important to consider during antenatal counselling of parents at risk of preterm delivery.^216^,^18^ Whether the impact of antenatal steroids is different between sexes is less clear cut, one study showing boys exhibiting a greater improvement in respiratory morbidity at gestations less than 29 weeks,^21^ others showing no difference.^20^ This study is the first to assess the impact of antenatal steroids on longitudinal growth while controlling for sex, gestational age at delivery and parental height.

Our study suggests there may be a difference between the sexes in the long-term effects of antenatal steroid exposure. While we have been able to control for gestational age and parental height, the study was underpowered to test for interaction effects between our covariates and the indicator variable of interest. Aforementioned studies^1 8^ have indicated that the long-term effects of antenatal steroids may differ depending on gestation at birth, and thus modelling larger data sets interacting exposure to steroids with gestational age as well as interacting sex with each covariate of interest and assessing for non-linear interactions would be of use. Testing such interactions within the data presented here would not be appropriate given the small sample size and high risk of overfitting. Nonetheless, our analysis provides a hypothesis of a sex-dependent effect of this treatment that warrants further investigation.

The larger size observed in girls in this cohort exposed to antenatal steroids may be mediated by the improved short-term benefits to lung, gut and brain health. Children who suffer from the complications of preterm birth more frequently associated with a lack of antenatal steroids are likely to have impaired growth due to some of these complications. As boys are at greater risk of neonatal complications after preterm birth, a reasonable hypothesis would be that they are more likely to exhibit a beneficial effect from treatment known to reduce these complications. Instead, we have seen the opposite association in terms of childhood growth, where a statistically significant effect was apparent only in girls.

The SITAR method has previously been employed to quantify the effect of birth weight and gestational age on later child growth patterns^26^ as well as improved trajectories of weight gain in preterm infants born in a contemporary cohort, likely due to improvements in neonatal care.^27^ This method allows for accurate estimation of individual and population-level growth curves that model variability in the timing and intensity of growth as well as size, appropriately accounting for the known variability in timing of the pubertal growth spurt. The extra ‘intensity’ of growth in girls exposed to antenatal steroids shown here tells us their larger ‘size’ was accompanied with faster growth in childhood in comparison to those not exposed, as opposed to a prolongation of growth.

The participants in this study exposed to antenatal steroids were born at an earlier gestation than those not exposed, and thus controlling for gestation within multivariable modelling has been important. Nonetheless, the observational cohort design is at risk of bias from unmeasured confounders such as socioeconomic status,^28^ and the cohort was of disproportionately white ethnicity. There was variability in the number of doses and timing of administration in those exposed to antenatal steroids. The single-centre observational design limits any assessment of postnatal steroid exposure due to confounding by indication, as infants treated with antenatal steroids are less likely to develop severe respiratory distress syndrome and thus less likely to require postnatal steroids. Neonatal care has advanced significantly since 1994 when this cohort was born, and thus the effect size we see here may not be applicable to a contemporary cohort. Neither magnesium sulphate nor delayed cord clamping for preterm infants was employed in clinical practice at the time of this study,^29 30^ although it is simpler not having to control for such covariates. The relatively small sample size and attrition of patients throughout the follow-up period have been accounted for using robust statistical techniques, but further research with larger cohorts is called for.

Healthy term infants in this study were not exposed to antenatal steroids, and thus this analysis cannot provide insights into the growth of infants that were exposed to antenatal steroids earlier in the pregnancy, but then went on to be born at term. Approximately 40%–50% of infants exposed to antenatal steroids fall into this category, and thus further research would be needed to understand any associations with growth in such patients, and further understand the increased associations with neonatal unit admission and long-term neurocognitive effects that currently have a low level of evidence.^8 9^

The administration of antenatal steroids to preterm infants is a well-documented event in obstetric care, and thus similar analyses could be carried out in other longitudinal cohorts to corroborate these findings. Power could be increased further by employing individual patient data meta-analysis.^31^ Following patients throughout childhood is challenging and resource intensive, but this study shows that by appropriately modelling height trajectories, we can gain insights into the dynamic process of growth beyond overly simplistic dichotomised outcome measures. Increasing understanding about the associations of medical interventions with the pattern of growth should help inform treatment strategies and antenatal counselling.

## Conclusion

This study finds a statistically and clinically significant greater mean final height among preterm girls exposed to antenatal steroids compared with those unexposed, an association not seen in boys. Analysis of other existing cohorts and long-term follow-up of neonates is indicated to further understanding of the impact of neonatal interventions on growth, to help inform treatment strategies and antenatal counselling.

## Supplementary material

10.1136/archdischild-2025-329091 online supplemental file 1

10.1136/archdischild-2025-329091 online supplemental material 1

## Data Availability

Data are available upon reasonable request.
