# Supplementary material for: Longitudinal modelling of growth in neonates exposed to antenatal steroids to quantify associations with final height: a cohort study
Source: Arch Dis Child. 2025 Jul 22;111(1):e329091. doi: 10.1136/archdischild-2025-329091 (PMC12772621; doi:10.1136/archdischild-2025-329091)
Supplement: online supplemental file 1 [file archdischild-111-1-s001.pdf]

Supplementary information

Supplementary appendix 1

Statistical methods

This study employs Superimposition by Translation and Rotation (SITAR) modelling to summarise repeated measures growth data. Measurements of growth are highly correlated within individual children, and thus the aim of modelling each curve is to extract important features of the non-linear nature of growth using a robust method of data reduction. The approach is to define an 'average' growth curve, and then estimate individual metrics to each patient that adjust the 'average' growth curve to fit an individual patient growth curve estimated by their data. The methodology employed is that of non-linear multilevel modelling, where the 'average' growth curve is defined by the model's 'fixed effects', and the individual patient growth curves defined by 'random effects' that are estimated for each patient.

Non-linear modelling

Non-linearity within SITAR is accounted for by fitting piecewise cubic splines through repeated measures data, different piecewise sections of the curve defined in between 'knots' on the x-axis. Each knot defines the proportion each basis function within the model contributes to the shape of the curve. The shape of the curve thus changes at each knot, but the change is gradual due to the contribution of different basis functions fading between sections, resulting in a smoothed curve. The number of knots defined within the model is set prior to model estimation by the number of degrees of freedom via the algorithm:

$$(n_{knots} = \text{degrees of freedom} - \text{degree of spline} - 1)$$

In the case of SITAR, cubic splines are employed, and the degree of spline is therefore 3. The knots are then positioned according to the quantiles of data throughout the age distribution, thus allowing for a greater proportion of knots at ages with more frequent measurements.

Multilevel modelling

The SITAR algorithm uses the package 'nlme' to estimate a non-linear model with fixed and random effects. The average non-linear spline model is fit through the data employing patient level translation along the y axis ('size'), translation on the x axis ('timing'), and scaling of the x axis ('intensity') to fit each individual patient growth curve to the individual patient data. This scaling of the x axis is compression or stretch of the average fixed effects fit across a central point of reference, which is geometrically

identical to a two-dimensional impression of the rotation of the curve around that central point of reference. The fixed and random effects parameters are then estimated using Restricted Maximum Likelihood which minimises the negative log-likelihood of the model given the available data and constraints upon the model fit. The resulting model will therefore have fixed effects defining the overall non-linear spline, and random effects of 'size', 'timing' and 'intensity' defined for each individual patient. Due to the sex differences in growth and pubertal development, boys and girls were modelled separately.

Model optimisation and fit statistics

The Bayesian Information Criterion (BIC) is a metric used to help define the most appropriate model fit. As the degrees of freedom are set prior to model estimation, this allows for penalisation of the model fit as the number of degrees of freedom increases, to avoid overfitting. The BIC is calculated by:

$$\text{BIC} = -2 \cdot (\ln \text{likelihood}_{\textit{full model}}) + n_{\textit{model parameters}} \cdot \ln n_{\textit{observations}}$$

The optimum model is then defined by the *lowest* BIC.

Each individual patient growth curve is used to calculate an error in y between the model fit and individual data points. These errors are squared, summed, averaged via their mean, and square rooted to achieve the root mean squared error of each model. This metric is on the same scale as the variable on the y axis (i.e. cm), and gives an average amount the individual patient growth curves deviate from the original height measurements.

Supplementary appendix 2

Attrition of patients throughout study

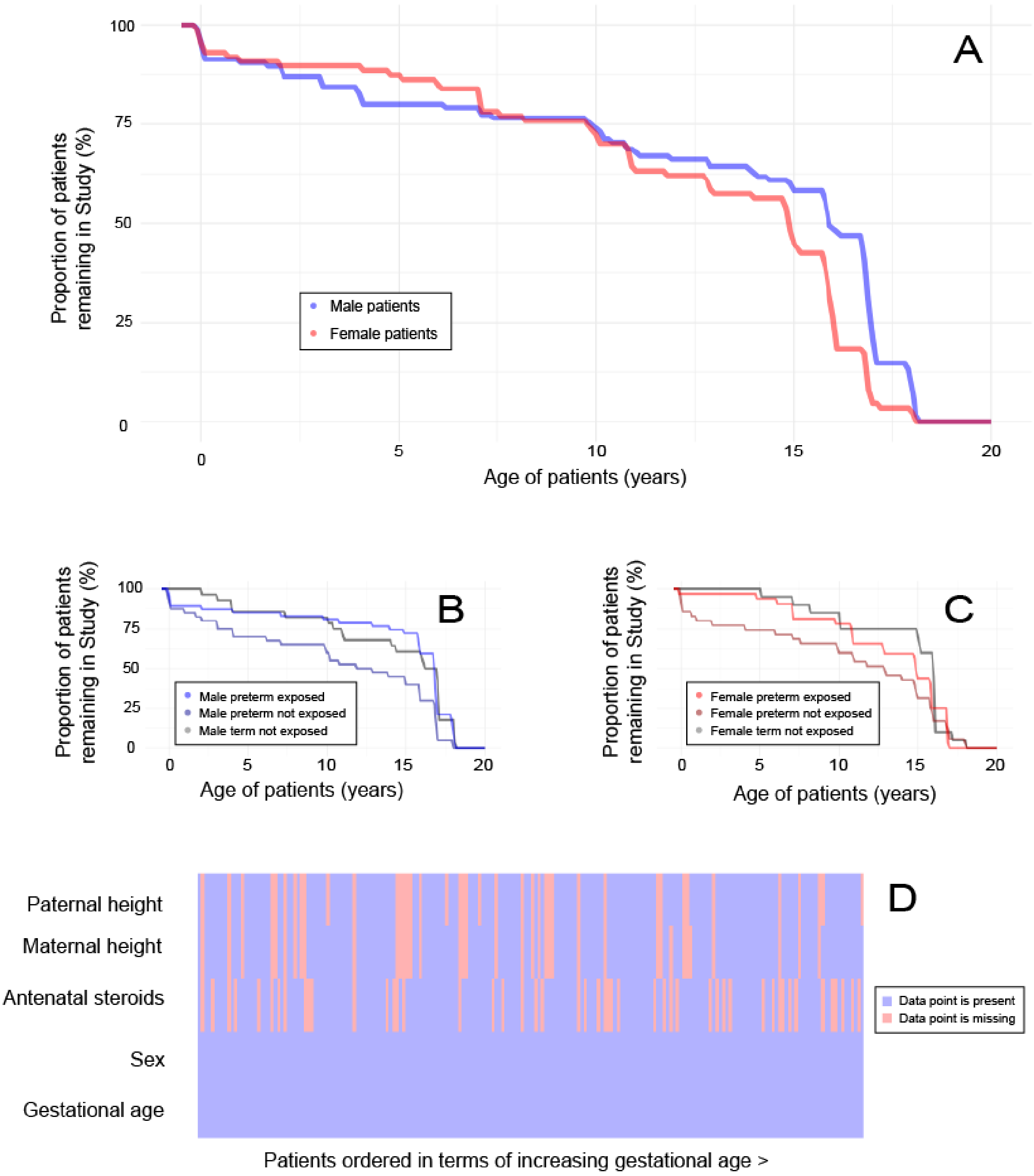

A: all participants separated by sex, B: male participants separated by antenatal steroid exposure, C: female participants separated by antenatal steroid exposure. There were similar levels of attrition between sexes, and between those exposed and not exposed to antenatal steroids. D shows distribution of missing data in covariates.

Supplementary appendix 3

Full model estimates adjusted for covariates

Boys

**Dependent Variable:**	***Independent Variables: Estimate (Standard error of the mean) p-value***					n in model (patients)	Model fit R^2 *(p-value)*
Raw data:	Intercept	Exposure to antenatal steroids: Yes	GA at delivery (weeks)	Maternal Height (cm)	Paternal Height (cm)		
Final measured height (cm)*	42.9 (34.7)	-7.958 (4.070) *p=0.065*	-0.184 (0.514) *p=0.725*	0.417 (0.236) *p=0.093*	0.427 (0.163) **p=0.017*	24	0.68 *(<0.001)*
SITAR calculated metrics							
Height at 18 years (cm)	69.2 (24.8)	-3.785 (2.558) **p=0.144*	-0.126 (0.273) *p=0.645*	0.381 (0.162) **p=0.022*	0.311 (0.115) **p=0.009*	66	0.36 *(<0.001)*
Peak pubertal height velocity (cm/year)	3.5 (3.4)	-0.519 (0.349) *p=0.142*	-0.049 (0.037) *p=0.195*	0.007 (0.022) *p=0.750*	0.037 (0.016) **p=0.023*	66	0.17 *(<0.001)*
Age at peak pubertal height velocity (years)	9.4 (3.5)	0.398 (0.366) *p=0.280*	0.049 (0.039) *p=0.212*	0.037 (0.023) *p=0.120*	-0.024 (0.016) *p=0.159*	66	0.07 *(0.365)*
SITAR patient level random effects:							
a: "Size"	-111.3 (18.0)	-1.028 (1.864) *p=0.583*	0.150 (0.199) *p=0.454*	0.466 (0.118) **p<0.001*	0.178 (0.084) **p=0.038*	66	0.44 *(<0.001)*
b: "Timing"	-1.4 (0.2)	0.017 (0.025) *p=0.509*	0.003 (0.003) *p=0.275*	0.007 (0.002) **p<0.001*	0.001 (0.001) *p=0.310*	66	0.38 *(<0.001)*
c: "Velocity"	-0.9 (0.2)	-0.018 (0.019) *p=0.338*	-0.001 (0.002) *p=0.565*	0.004 (0.001) **p=0.004*	0.002 (0.001) **p=0.007*	66	0.40 *(<0.001)*

Girls

**Dependent Variable:**	***Independent Variables: Estimate (Standard error of the mean) p-value***					n in model (patients)	Model fit R^2 (p-value)
Raw data:	Intercept	Exposure to antenatal steroids: Yes	GA at delivery (weeks)	Maternal Height (cm)	Paternal Height (cm)		
Final measured height (cm)*	18.1 (21.9)	2.688 (1.955) *p=0.178*	0.229 (0.213) *p=0.291*	0.292 (0.089) **p=0.003*	0.498 (0.106) **p<0.001*	39	0.57 *(<0.001)*
SITAR calculated metrics							
Height at 18 years (cm)	41.3 (15.0)	2.765 (1.250) **p=0.031*	0.370 (0.136) **p=0.009*	0.310 (0.060) **p<0.001*	0.330 (0.136) **p<0.001*	59	0.56 *(<0.001)*
Peak pubertal height velocity (cm/year)	-1.3 (3.2)	0.350 (0.264) *p=0.190*	-0.028 (0.029) *p=0.333*	0.030 (0.013) **p=0.021*	0.028 (0.014) **p=0.047*	59	0.24 *(<0.001)*
Age at peak pubertal height velocity (years)	12.6 (4.1)	-0.139 (0.341) *p=0.686*	0.053 (0.037) *p=0.163*	-0.006 (0.016) *p=0.723*	-0.013 (0.018) *p=0.459*	59	0.08 *(0.310)*
SITAR patient level random effects:							
a: "Size"	-121.5 (15.1)	2.871 (1.256) **p=0.026*	0.369 (0.137) **p=0.010*	0.317 (0.061) **p<0.001*	0.314 (0.065) **p<0.001*	59	0.55 *(<0.001)*
b: "Timing"	-1.2 (0.3)	0.030 (0.025) *p=0.229*	0.005 (0.003) *p=0.083*	0.003 (0.001) **p=0.010*	0.003 (0.001) **p=0.034*	59	0.24 *(<0.001)*
c: "Velocity"	-1.1 (0.1)	0.035 (0.012) **p=0.006*	0.001 (0.001) *p=0.550*	0.003 (0.001) **p<0.001*	0.003 (0.001) **p<0.001*	59	0.55 *(<0.001)*
