# Supplementary material for: Longitudinal modelling of growth in neonates exposed to antenatal steroids to quantify associations with final height: a cohort study
Source: Arch Dis Child. 2025 Jul 22;111(1):e329091. doi: 10.1136/archdischild-2025-329091 (PMC12772621; doi:10.1136/archdischild-2025-329091)
Supplement: online supplemental material 1 [file archdischild-111-1-s002.pdf]

Longitudinal modelling of growth in neonates exposed to antenatal steroids to quantify associations with final height: a cohort study

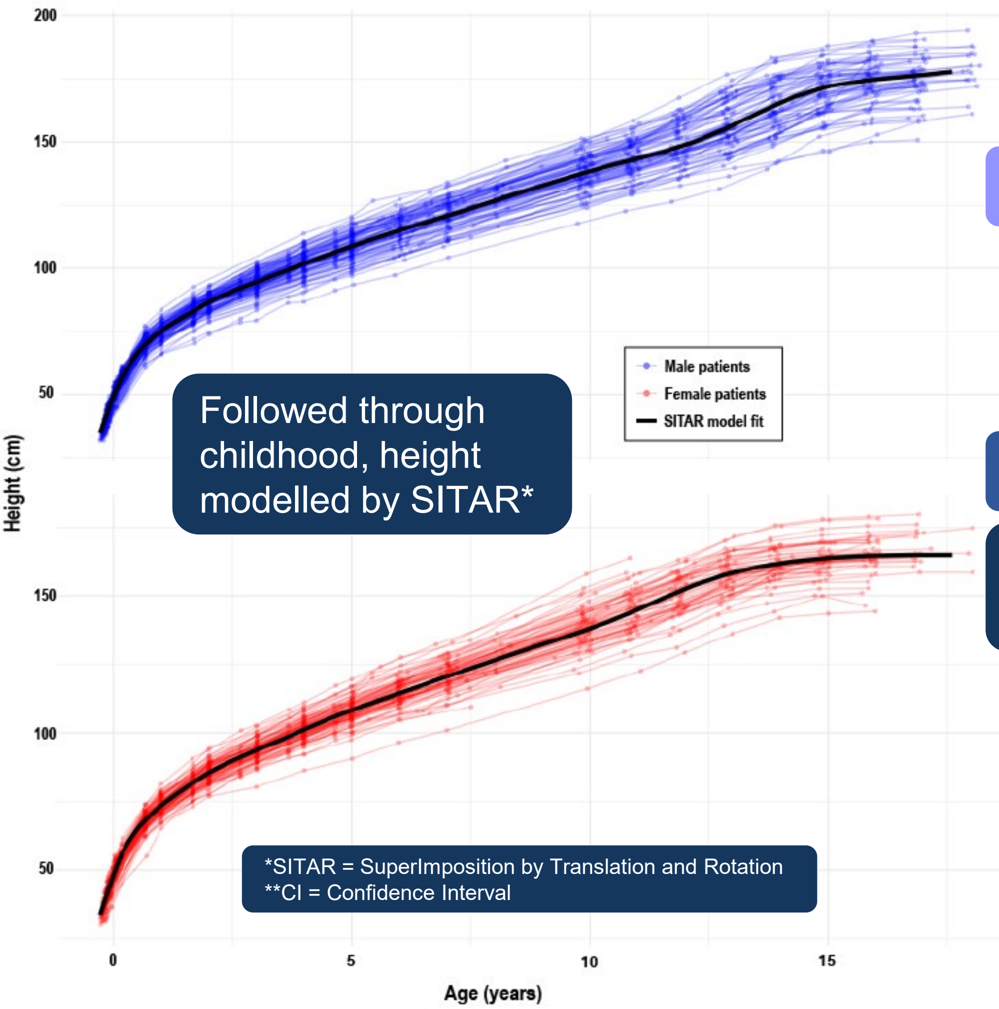

Followed through childhood, height modelled by SITAR*

*SITAR = SuperImposition by Translation and Rotation
**CI = Confidence Interval

Observational cohort study following growth of preterm infants and term controls

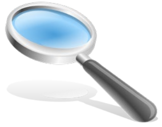

27 male babies not exposed to antenatal steroids

32 female babies not exposed to antenatal steroids

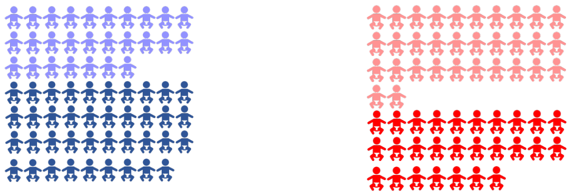

39 male babies exposed to antenatal steroids

27 female babies exposed to antenatal steroids

SITAR outcome metrics modelled on exposure to antenatal steroids, adjusting for gestational age and parental height

Greater height at 18 years in girls exposed to antenatal steroids than girls not exposed

2.8cm (95% CI** 0.3 to 5.3cm)

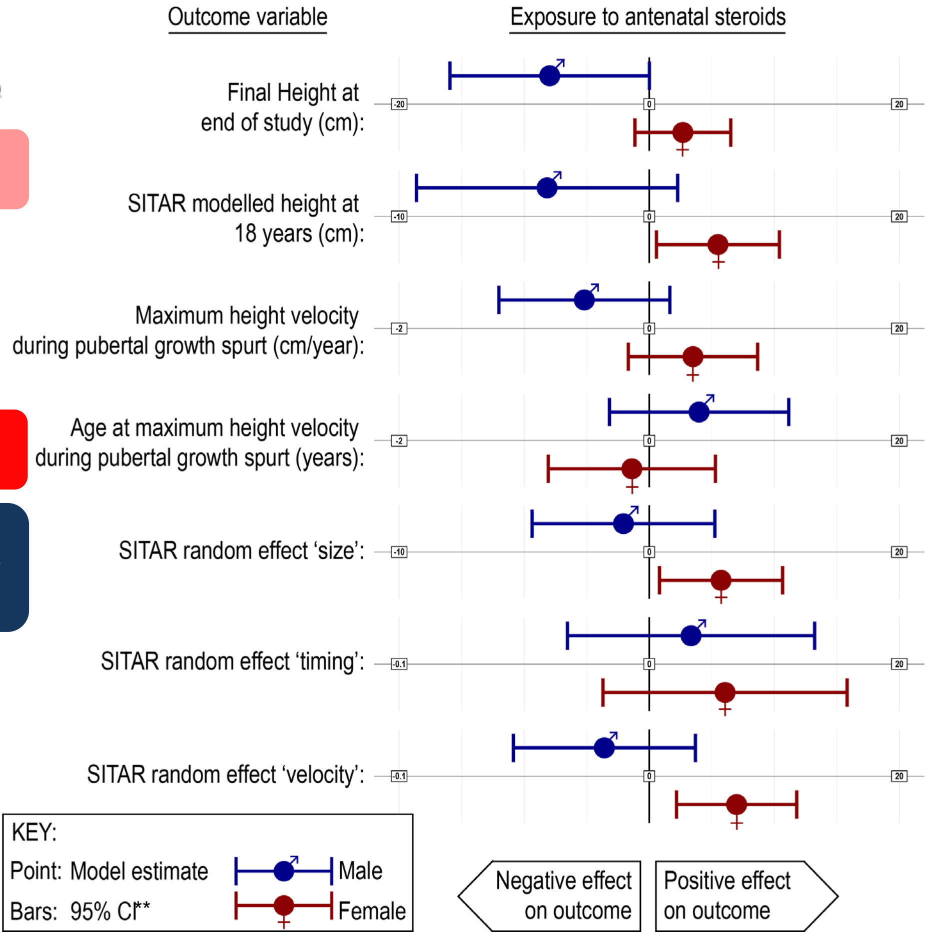

BMJ Group

RCPCH Royal College of Paediatrics and Child Health Leading the way in Children's Health

adc.bmj.com fn.bmj.com ep.bmj.com

Archives of Disease in Childhood
